# Resuscitation Following a Bupivacaine Injection for a Cervical Paravertebral Block

**DOI:** 10.1515/med-2019-0112

**Published:** 2019-12-17

**Authors:** Saulius Vosylius, Valentinas Uvarovas, Saulė Svediene, Igoris Satkauskas

**Affiliations:** 1 Clinic of Anaesthesiology and Intensive Care, Faculty of Medicine, Vilnius University, Republican Vilnius University Hospital, Siltnamiu 29, Vilnius, LT-04130, Vilnius, Lithuania; 2 Clinic of Rheumatology, Orthopaedic Traumatology and Reconstructive Surgery, Faculty of Medicine, Vilnius University, Siltnamiu 29, Vilnius, LT-04130, Vilnius, Lithuania

**Keywords:** Local anaesthetic, Ventricular fibrillation, Cardiopulmonary resuscitation, Fat emulsions, Intravenous

## Abstract

**Background:**

Cardiac arrest related to nerve blockade using a local anaesthetic is a rare event. We report a case of bupivacaine severe cardiovascular toxicity following cervical paravertebral nerve block.

**Case presentation:**

A 44-year-old female was admitted to Republican Vilnius University Hospital, with symptoms of sustained severe pain in her neck that radiated to both arms. Multiple cervical intervertebral hernias with spinal stenosis were confirmed by magnetic resonance imaging. Following infiltration of the subcutaneous tissue with a 0.5 % bupivacaine solution, an 18-gauge spinal needle was used to perform the paravertebral block at the C6 level. Bupivacaine was injected in incremental doses to a total of 10 mL. Rapid loss of consciousness and cardiovascular collapse suggested a neuraxial injection of bupivacaine. Long-lasting cardiopulmonary resuscitation, including chest compressions, defibrillation attempts for refractory ventricular fibrillation, medications, mechanical ventilation, and intravenous lipid emulsion infusion, was successful. No severe adverse outcomes other than acute kidney injury and chest pain related to prolonged chest compressions were documented.

**Conclusions:**

This case report emphasizes the necessity of ensuring adequate safety precautions to avoid local anaesthetic systemic toxicity. Lipid emulsion preparations should be available in all hospital settings where local anaesthetics are used for regional anaesthesia or pain management.

## 1 Introduction

Local anaesthetic systemic toxicity (LAST)-related events range from mild symptoms to serious central nervous system and cardiovascular presentations. Life-threatening complications during cervical paravertebral block are not unusual. Numerous structures (vertebral, carotid, and jugular major vessels, as well as the phrenic nerve) are close to the nerve roots in the neck; thus local anaesthetic (LA) injection can be associated with a range of complications. Lipophilic LAs such as bupivacaine, ropivacaine, or levobupivacaine are most commonly used. Bupivacaine, a long-acting highly lipophilic LA agent, has been used for pain control in both ambulatory and hospitalized patients.

This article reports the successful resuscitation with full neurological recovery after cardiac arrest resulting from prolonged ventricular fibrillation provoked by inadvertent central neuraxial injection of LA during a cervical paravertebral block procedure. Cardiac arrest related to nerve blockade is a rare event: cardiac arrest has been reported in only 15% (17 of 114) of LAST case reports from 2010 to 2016 [1, 2]. A systematic review of the literature through the end of 2016 found 18 case reports of cardiac arrest when intravenous lipid emulsion (ILE) was used to manage LAST [3].

## 2 Case presentation

**Figure 1:**
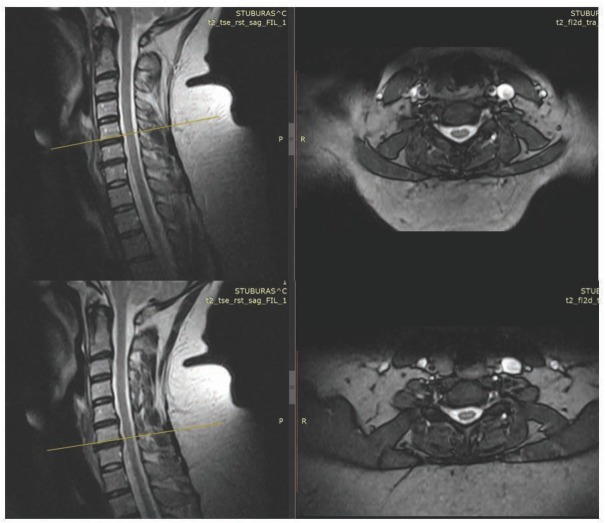
Magnetic resonance imaging of the cervical spine shows intervertebral hernias in C5-C6, C6-C7, and cervical spinal stenosis.

A 44-year-old female was admitted to the orthopaedic department at the Republican Vilnius University Hospital on August 3, 2018; her symptoms were sustained severe pain at 8 to 9 points on a verbal analogue scale in the neck that radiated to both arms, along with feelings of weakness in the arms. The significant aspects of the patient’s medical history were obesity (weight 126 kg, body mass index (BMI) 40.7 kg/m^2^) and arterial hypertension. She had been taking non-steroid analgesics, nebivolol, and perindopril prior to admission to the hospital. Hyperesthesia was found on left C5, C6 and right C4, C5, C6 with normal muscle strength. Intervertebral hernias in C2-C3, C5-C6, C6-C7, cervical spinal stenosis, and C2 retrolisthesis were confirmed by magnetic resonance imaging (Fig. 1).

The patient was fitted for a brachial plexus block at the cervical paravertebral approach. In the radiology department; she was placed on her right side, and a blockade on the left side of the neck was performed under aseptic conditions by an experienced orthopaedic surgeon following confirmatory X-ray. Standard monitoring was applied, including electrocardiography (ECG), pulse oximetry, and blood pressure measurement. After a thorough infiltration of the subcutaneous tissue with 0.5% bupivacaine hydrochloride solution, an 18-gauge Whitacre-tip spinal needle was used to perform the cervical paravertebral block at the C6 level. The posterior approach was chosen to avoid puncturing blood vessels, and primarily, to reach the sensory fibers of the roots. The needle was introduced at the apex of the V-shaped space formed by the trapezius and levator scapulae muscles 6 cm laterally from the spinous process. Sustaining a slightly anterior, medial, and caudal direction, the needle progressed until contact with the transverse process of C6 at a depth of 5 cm from the skin. Following a negative blood aspiration test, 0.5% bupivacaine was injected in incremental doses over several minutes to a total of 10 mL. Two minutes later, the patient began to exhibit paraesthesia in the arms and became confused, then apnoeic and unresponsive. Pulseless cardiac electrical activity with sinus bradycardia developed. Cardiopulmonary resuscitation (CPR) was initiated using chest compressions and bag-mask ventilation. The patient underwent endotracheal intubation when a resuscitation team arrived. The return of spontaneous circulation was achieved after four minutes. Arterial blood pressure was measured at 60⁄40 mmHg with a sinus rhythm at 125 beats per minute. Using a portable ventilator and observing vital signs on the monitor, the patient was transported to the intensive care unit (ICU), and volume control mandatory ventilation was then performed. With a presumptive diagnosis of bupivacaine systemic toxicity, a 100 mL bolus of Lipofundin 20% (B. Braun, Melsungen AG, Germany) was administered, followed by subsequent continuous infusion of 400 mL over 30 minutes. CPR was resumed with monophasic defibrillation 360J without delay when ventricular fibrillation occurred 23 minutes after the first cardiopulmonary collapse. Uninterrupted chest compressions were performed except during the assessment of cardiac rhythm and the defibrillation attempt. Repeated defibrillation attempts were required ten times for sustained ventricular fibrillation. Adrenaline 1 mg was administered every 4-5 min. for a total dose of 10 mg. Amiodarone 300 mg was given after three defibrillation attempts with an additional 150 mg later on. After 47 minutes of CPR, the patient returned to spontaneous circulation, and the sinus rhythm was restored. We observed that the patient was making purposeful movements and opened her eyes while cardiac arrest persisted. When spontaneous circulation returned, ECG demonstrated ST depression in leads I, II, aVL, and V2-V6. Noradrenaline was administered continuously to maintain haemodynamic stability at a maximal dose of 0.3 μg/kg/min. The patient was sedated with midazolam (1 mg/h) and fentanyl (0.16 mg/h) infusion to an appropriate level of consciousness (Richmond Agitation Sedation Scale up to -3). A transthoracic echocardiogram showed an absence of abnormal motions in the heart wall, an ejection fraction of 40%, and minor dilatation of all chambers with regurgitation through the mitral and tricuspid valves. Blood test results shortly after the return of spontaneous circulation were abnormal, showing high sensitivity troponin I 31,004.9 ng/L [normal range < 2 ng/L], D-dimer 35,500 μg/mL [normal range < 200 μg/L], urea 9.49 mmol/L [normal range 2.10-8.30 mmol/L], lactate 12.9 mmol/L [normal range 0.5–1.6 mmol/L], serum creatinine 127 μmol/L [normal range 44-106 μmol/L], and glucose 21.63 mmol/L [normal range 3.9-5.8 mmol/L]. The next morning, haemodynamic stability was achieved, and high sensitivity troponin I was 18,748.6 ng/L. The patient had full consciousness and was subsequently disconnected from the ventilator and extubated. Non-oliguric acute kidney injury developed, serum creatinine increased up to 402 μmol/L with urea to 21.17 mmol/L, and continuous furosemide infusion was required. Six days later, the patient was transferred from the ICU to the ward at the orthopaedic department. The patient had complaints of dizziness, decreased hearing, and pain in the chest wall but did not sustain any significant neurological or cardiovascular deficits. Serum urea and creatinine values returned to normal ranges on seventh day. After an additional two weeks of observation, the patient was discharged from the hospital.

**Ethical approval**: The research related to human use has been complied with all the relevant national regulations, institutional policies and in accordance the tenets of the Helsinki Declaration, and has been approved by the authors’ institutional review board or equivalent committee.

**Informed consent**: Informed consent has been obtained from the patient included in this study.

## 3 Discussion and conclusions

LAST usually occurs in a hospital’s operating theatre area as a result of local tissue infiltration, or peripheral nerve or centro-neuraxial block procedures. Anaesthesiologists and surgeons comprise the majority of physicians who use long acting LA injection for a wide range of indications. Lipophilic LAs such as bupivacaine, ropivacaine or levobupivacaine are preferred for their potency and duration of action but carry the risk of developing LAST in the event of inadvertent intravascular injection or excessive systemic absorption from tissues. The paravertebral nerve block approach is usually used to treat acute or chronic pain and also for surgeries involving cervical, thoracic and lumbar dermatomes. We report a case of cardiac arrest after bupivacaine injection in an adult patient undergoing cervical paravertebral brachial plexus block. Long-lasting CPR with ILE infusion resulted in haemodynamic nor alisation after cardiac arrest with persistent ventricular fibrillation. No severe adverse outcomes other than acute kidney injury and chest pain related to the prolonged chest compressions were documented.

A systematic review of recent large clinical studies including 20,021 patients who received peripheral nerve blockade found a low incidence of mild signs of LA systemic toxicity, which was even lower when ultrasound guidance was used, with only one incident that progressed to cardiac arrest [4]. A prospective study following paravertebral nerve block in 620 adult patients showed that the most common complications were inadvertent vascular puncture, arterial hypotension, and epidural or intrathecal spread. An overall failure rate was found to be 6.1% [5].

In this case, there was a rapid loss of vital functions suggesting central neuraxial injection of bupivacaine. However, negative blood aspiration prior to the injection does not exclude the possibility of intravascular needle placement. The high troponin level after the return of spontaneous circulation suggested global coronary hypoperfusion and myocardial injury. We did not observe tonic-clonic seizures after the bupivacaine injection or during resuscitation. Three reviews of reported LAST events over almost four decades found 207 cases with a variable pattern of clinical presentations [1, 2, 7]. The classic cascade of systemic toxicity begins with prodromal signs and central nervous system symptoms from excitation to seizure and coma, and subsequent cardiovascular collapse predominates [6]. The atypical LAST presentations were reported at a higher rate in case reports describing combined central nervous system and cardiovascular or isolated cardiovascular signs and symptoms. Among all these cases reports from 1979 to 2016, 14% (30 of 207) of the patients exhibited signs of cardiovascular toxicity alone [1, 2, 7]. A collaborative lipid emulsion workgroup established by the American Academy of Clinical Toxicology to review the evidence on the effect of intravenous lipid emulsion for LA toxicity found 73 case reports including 83 patients. The most common presentations of LA toxicity were central nervous system depression or agitation (54%), seizures (59%), hypotension, arrhythmias (47%), cardiac arrest (22%), CPR and a requirement for endotracheal intubation or/and mechanical ventilation (42%) [3].

The patients are considered to be at higher risk for LA toxicity if they have pre-existing cardiac, liver, metabolic, or neurologic disease, low plasma protein, small body mass or at the extremes of age [9]. LAs have different cardio-toxic effects. Bupivacaine, levobupivacaine, and ropivacaine are negatively inotropic and arrhythmogenic, whereas lidocaine and mepivacaine decrease contractility [8]. Bupivacaine has been considered to be the most cardio-toxic compared with the other LAs and is most difficult to resuscitate when systemic toxicity occurs [6, 9]. There are no published data that describe an optimal dose or concentration of LA for either single-shot injection or continuous paravertebral infusion. Longer-acting, relatively diluted bupivacaine (0.25 to 0.5%) is preferred for paravertebral blocks. In adults, the commonly used bolus dose for thoracic paravertebral block is 20 mL 0.5% bupivacaine and results in the maximum plasma concentration in a median time of 25 min (range, 10-60 min) [10]. The upper toxicity threshold limits are considerably variable, but a bupivacaine dose of 50 mg and a diminished volume up to 10 mL for a cervical level block in our patient with high body weight seemed to be inconsistent with the risk of significant intoxication due to excessive systemic absorption.

The management of LAST includes supportive treatment to control seizures and maintain circulation. In recent guidelines, the recommendation to use ILE during advanced cardiac life support after cardiac arrest due to bupivacaine toxicity is based on the results of systematic reviews and an expert consensus [11]. The level of evidence is high; the risk/benefit ratio in severe systemic toxicity favours the use of ILE and is believed to involve multiple mechanisms. Lipid help to move LA out of cardiac tissue and brain to muscle where it is stored and liver where its metabolism, improve myocardial contractility, cardiac output, blood flow and blood pressure through actions on the vasculature and heart, and provide a post-conditioning cardio-protective effects [12]. Data are lacking with regard to ILE dose – response relationships for treating bupivacaine toxicity. The recommendation is neutral regarding the choice of ILE dosing and the endpoints to initiate or stop ILE administration; the most commonly reported dosing regimen is a bolus of 1.5 mL/kg and an infusion of 0.25 mL/kg/min of a 20% lipid emulsion [11]. The Association of Anaesthetists of Great Britain and Ireland (AAGBI) recommended an initial bolus of 1.5 mL/kg lipid emulsion 20% administered over 1 minute, and this dose may be repeated twice at 5-minute intervals if adequate circulation has not been restored, following an infusion of 15 mL/kg/h [13]. The last update of the recommendations from the American Society of Regional Anaesthesia and Pain Medicine for patients with a body weight above 70 kg (ideal body weight) suggest 100 mL lipid emulsion of 20% following an infusion of 200-250 mL over 15-20 min. In the case of circulatory instability, a re-bolus or increased infusion to 0.5 mL/kg/min should be considered [9]. When ventricular fibrillation is persistent, the lipid bolus must be repeated or the infusion rate can be increased because of bupivacaine’s high protein binding (95%), lipophilicity, and subsequent hypercapnia, acidosis and hypoxemia during long resuscitation efforts. In our case, the injected dose of bupivacaine did not suggest overdosing, and the total ILE dose used to treat LAST was lower than recommended in the guidelines. Due to the limitation that we could not measure plasma bupivacaine concentrations, we are unable to comment on the effect of lipid emulsion on bupivacaine serum concentrations.

Prolonged resuscitation efforts were performed with continued high-quality chest compressions, with minimal interruption for a defibrillation attempt upon request to change the person performing the chest compressions. Spontaneous limb movements and eye opening suggesting clinical signs of life were observed during CPR. The next day, the patient had an awareness of being alive and memories during the resuscitation. High-quality chest compressions and ventilation can generate sufficient circulation and brain oxygenation to restore signs of life [14]. CPR-induced consciousness has been described in varying levels, ranging from mild eye opening and breathing to active interference of the rescuers’ CPR attempts. The true incidence of CPR-induced consciousness remains unknown. In the prospective AWARE trial, more than half (55 out of 101) survivors of a cardiac arrest who were able to complete the interviews had self-reported perceived awareness or memories [15].

Increased understanding among anaesthesiologists and other physicians of the dangers of LAST has led to the introduction of safety measures and recommendations regarding the maximum dosage [6]. We recognize that it was unsafe practice to perform paravertebral block without ultrasound guidance. Ultrasound has been introduced to reduce the risk of LAST because of its ability to visualize the needle and anatomic structures, increasing the accuracy of LA delivery and thus reducing the incidence of vascular injection. However, this case suggests the necessity for more safety precautions during interventions involving LAs, including ultrasound-guided nerve block, the necessity of frequent needle aspiration before LA injection, fractionated administration of LA, the use of pharmacologic markers or a test dose, continuous vital sign monitoring and the presence of well-trained personnel for early recognition and immediate management of LAST.

The American Society of Regional Anaesthesia and Pain Medicine recognizes that the pharmacological treatment of LAST is different from other cardiac arrest scenarios and recommends smaller boluses (≤ 1 mcg/kg) of adrenaline than typical for cardiac arrest [16]. The availability of ILE infusion is crucial in settings where nerve blocks with LA are performed. Regardless of branded preparation, 20% lipid emulsion should be available in both inpatient and outpatient settings where LAs are routinely used. Lipid rescue for presumed LA toxicity should be considered during at the first clinical signs after airway management [9]. The use of ILE must be initiated in any LAST event that is judged to be potentially serious.

In conclusion, we report a rare case of bupivacaine systemic toxicity while performing cervical paravertebral nerve block in a hospital setting. Long-lasting CPR including high-quality chest compressions, defibrillation attempts for refractory ventricular fibrillation, medications, and mechanical ventilation with lipid rescue treatment was successful. More widespread awareness of the potential effectiveness of ILE infusion immediately after presumably recognized bupivacaine cardiac toxicity could contribute to successful CPR; 20% lipid emulsion preparations should be immediately available in all hospital settings where LA are used for regional anaesthesia or pain management.

## Abbreviations

LA: local anaesthetic
LAST: local anaesthetic systemic toxicity
ECG: electrocardiography
CPR: cardiopulmonary resuscitation
ILE: intravenous lipid emulsion
